# RNAi knockdown of acetyl-CoA carboxylase gene eliminates jinggangmycin-enhanced reproduction and population growth in the brown planthopper, *Nilaparvata lugens*

**DOI:** 10.1038/srep15360

**Published:** 2015-10-20

**Authors:** Yi-Xin Zhang, Lin-Quan Ge, Yi-Ping Jiang, Xiu-Li Lu, Xin Li, David Stanley, Qi-Sheng Song, Jin-Cai Wu

**Affiliations:** 1 School of Plant Protection, Yangzhou University, Yangzhou, 225009, P.R.China; 2 USDA/Agricultural Research Service, Biological Control of insect Research Laboratory, Columbia, Missouri; 3 Division of Plant Science, University of Missouri, Agriculture Building, Columbia, MO 65211, USA

## Abstract

A major challenge in ecology lies in understanding the coexistence of intraguild species, well documented at the organismal level, but not at the molecular level. This study focused on the effects of the antibiotic, jinggangmycin (JGM), a fungicide widely used in Asian rice agroecosystems, on reproduction of insects within the planthopper guild, including the brown planthopper (BPH) *Nilaparvata lugens* and the white-backed planthopper (WBPH) *Sogatella furcifera*, both serious resurgence rice pests. JGM exposure significantly increased BPH fecundity and population growth, but suppressed both parameters in laboratory and field WBPH populations. We used digital gene expression and transcriptomic analyses to identify a panel of differentially expressed genes, including a set of up-regulated genes in JGM-treated BPH, which were down-regulated in JGM-treated WBPH. RNAi silencing of *Acetyl Co-A carboxylase* (*ACC*), highly expressed in JGM-treated BPH, reduced *ACC* expression (by > 60%) and eliminated JGM-induced fecundity increases in BPH. These findings support our hypothesis that differences in *ACC* expression separates intraguild species at the molecular level.

A major challenge in ecology is understanding the coexistence of intraguild species^1^. Guild studies mainly focus on the community assemblages and species interactions^2^,^3^,^4^ and the effect of intraguild predation on community organization or competition^5^,^6^,^7^,^8^. Knowledge of specific adaptations to environmental factors at the molecular level within intraguild species is lacking, particularly in agroecosystems, in which the main research thrust is focused on the influence of pesticides. Pesticides are powerful operators within agroecosystems, responsible for pest resistance to applied chemicals, for pest resurgence and for many deleterious non-target effects. Research into the molecular mechanisms of pesticide actions and the evolution of resistance to pesticides has yielded extensive knowledge of these processes, however, molecular aspects of differential pesticide actions within intraguild species remains a substantial knowledge gap.

The total pesticide load in agroecosytems includes herbicides, fungicides and insecticides. Pesticide actions go beyond lethality of target species. Insecticides exert substantial sublethal effects on beneficial arthropods, such as influencing learning and behavior^9^. More to the point, herbicides and fungicides impact insect biology. For example, exposure to very low levels of the herbicide, paraquat, reduced the sizes of honeybee larval oenocytes, a class of hemocytes^10^. These cells act in insect immunity, and exposure to an herbicide can lead to the non-obvious effect of suppressing insect immunity at the cellular level. New knowledge is required to predict the influences of agricultural chemicals on the complex interactions among species, within and outside of guilds.

Members of the planthopper guild, including the brown planthopper (BPH) *Nilaparvata lugens* Stål, the white back planthopper (WBPH) *Sogatella furcifera* Horvath, and the small brown planthopper (SBPH) *Laodelphax striatella* Fallén, are serious rice pests^11^,^12^,^13^. Some, but not all, pesticides stimulate BPH reproduction^14^,^15^,^16^, emphasizing the current inability to predict in advance how agricultural chemicals will influence agro-ecosystems.

Rice sheath blight is responsible for substantial crop losses, which are partly alleviated with fungicides, including the antibiotic jinggangmycin (JGM), a product of *Streptomyces hygroscopicus* var. *jinggangensis*^17^. JGM foliar spray stimulates BPH fecundity, increases survival rate of nymphs and promotes the occurrence of hopper burn, but suppresses WBPH reproduction in laboratory experiments^18^,^19^. There is no information on the molecular mechanisms of JGM-induced changes in BPH and WBPH reproduction. The planthopper guild has been exposed to JGM, applied two or three times each growing season at commercial (or greater) rates, for over 40 years^17^. The BPH and WHPH have evolved inverse responses to JGM, a constant chemical feature of their environment. These responses follow separate molecular adaptations to JGM, which may facilitate their sharing of very similar niches. We conceive of this process as molecular niche separation (MNS), which we pose here as a hypothesis. In this paper we report on the outcomes of our molecular explorations designed to test our hypothesis.

## Results

### JGM exerts contrasting effects on natural BHP and WBPH populations

**Figure 1:**
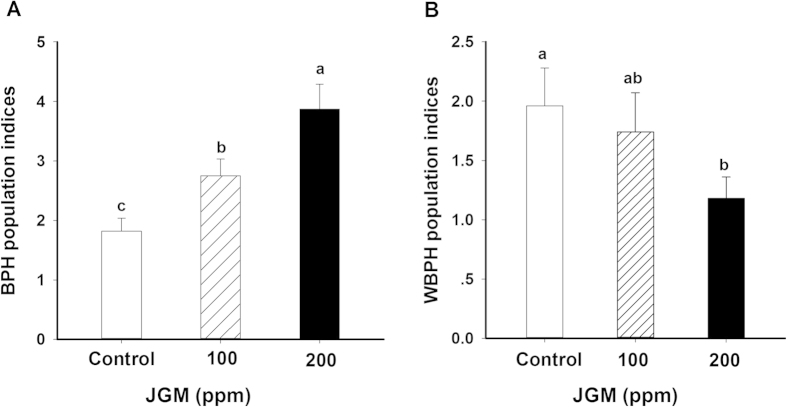
JGM foliar spray treatments (at 100 and 200 ppm) influenced planthopper populations in rice fields. Control was sprayed with solvent [tap water and non-active substances (dimethyl sulfoxide and emulsifier) that lacked the effects of insecticides]. The histogram bars represent mean ± SEM of ≥4 biological replicates; bars annotated with the same letter are not significantly different at *p *< 0.05. Panel A: BPH indices. Panel B: WBPH indices.

Data on field populations verified the significant effects of JGM on BPH (↑42% at 100 ppm and ↑99% at 200 ppm) and WBPH (↓12% at 100 ppm and ↓71% at 200 ppm), compared to controls (Fig. 1) (Table 1).

### Analysis of JGM-induced gene expression profiles

**Figure 2:**
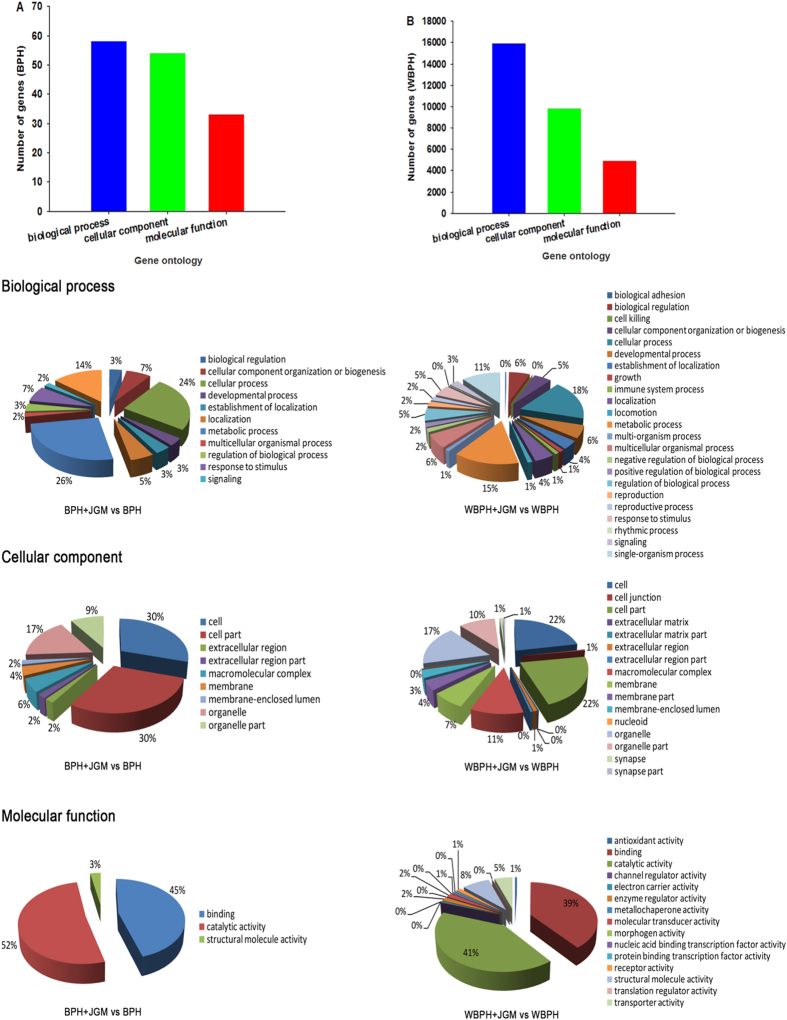
DGE and transcriptome analysis of differentially expressed genes in JGM treated and control (solvent only) BPH and WBPH. Median-normalized expression levels sorted by fold-change (upregulated FDR ≤ 0.001, log 2 Ratio ≥ 1; down regulated FDR ≤ 0.001, log 2 Ratio ≤ −1). GO analysis was performed using the WEGO software.

**Figure 3:**
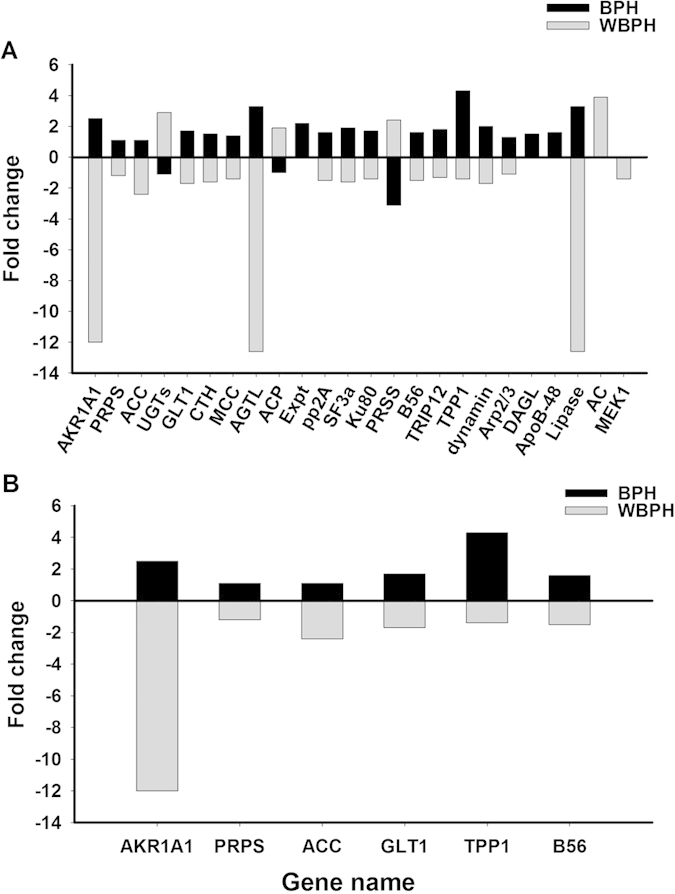
Genes inversely expressed in BPH and WBPH after JGM treatment (A) and genes inversely expressed, associated with reproduction and metabolism (B). Histogram bars indicate extent of change, with the x-axis set at zero. Bars above the x-axis represent up-regulation and bars below represent down-regulation. Abbreviations of all genes: *AKR1A1* = *alcohol dehydrogenase (NADP+*); *PRPS* = *ribose-phosphate pyrophosphokinase*; *ACC* = *acetyl-CoA/propionyl-CoA carboxylase*; *GLT1* = *glutamate synthase (NADPH/NADH*); *CTH* = cystathionine gamma-lyase; *MCC *= *3-methylcrotonyl-CoA carboxylase alpha subunit*; *AGT L* = *triacylglycerol lipase*; *ACP* = *acid phosphatase*; *Expt* = *exportin-T*; *pp 2A* = *serine/threonine-protein phosphatase 2 A catalytic subunit*; *SF3a* = s*plicing factor 3A subunit 1*; *Ku 80* = *ATP-dependent DNA helicase 2 subunit 2*; *PRSS* = *protease serine*; *TRIP 12* = *E3 ubiquitin-protein ligase TRIP12*; *TPP 1* = *tripeptidyl-peptidase I*; *B56* = *serine/threonine-protein phosphatase 2 A regulatory subunit B*; *dynamin* = *dynmin GTPase*; *Arp 2/3* = *actin related protein 2/3 complex, subunit 5*; *DAGL* = *sn1-specific diacylglycerol lipase*; *ApoB-48* = *apolipoprotein B*; *Lipase* = *gastric triacylglycerol lipase*; *AC* = *adenylate cyclase 1*; *MEK1* = *mitogen-activated protein kinase kinase 1.*

Digital gene expression (DGE) analysis showed that 145 genes were differentially expressed (85↑and 60↓) between JGM-treated and untreated control BPHs while transcriptomic analysis showed that 15,870 genes were differentially expressed (10,761↑ and 5,109↓) between JGM-treated WBPH and untreated control WBPH. We ascribe the large difference in number of influenced genes to the two different approaches we had to use in this study (see M&M). Analysis of the molecular function, biological process, and cellular component of gene products by gene ontology (GO) showed for BPH, 58 differentially expressed genes were assigned to “Biological Process” including metabolic process (26%), cellular process (24%); 54 differentially expressed genes were placed in “Molecular Function” including catalytic activity (52%), binding (45%) and structural molecule activity (3%); and 33 differentially expressed genes were assigned to “Cellular Component” including cell part (30%), cell (30%) and others (40%) (Fig. 2). For WBPH, 15,902 differentially expressed genes were assigned to “Biological Process”, 9,830 differentially expressed genes into “Molecular Function”, and 4,855 differentially expressed genes into “Cellular Component” (Fig. 2). Comparing JGM-treated BPH and WBPH, expression of 24 genes was inversely changed (Fig. 3A), six of them associated with reproduction and energy metabolism (Fig. 3B), all selected for this figure because of their inverse responses to JGM in the two planthopper species. We targeted *Acetyl Co-A Carboxylase* (*ACC*) for functional analysis of the JGM influence on BPH and WBPH reproduction because the basal level of *ACC* expression in controls was relatively high (20.07 in treatment vs 9.13 in control for BPH; 0.81 in treatment vs 4.18 in control for WBPH) and because it is related to energy metabolism required for reproduction.

### The influence of *ACC* silencing on planthopper reproduction

**Figure 4:**
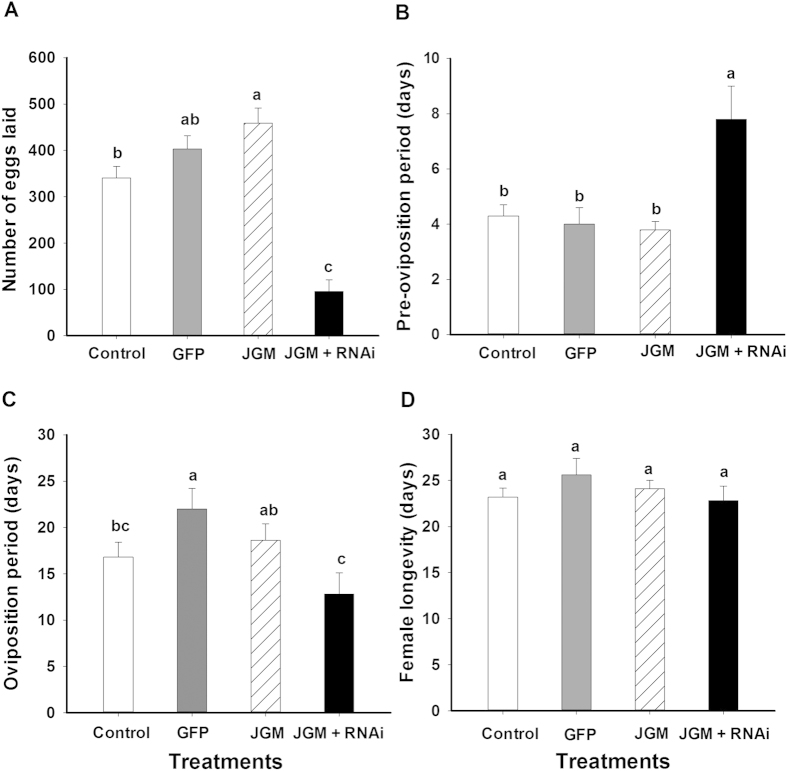
Dietary dsACC influenced BPH reproduction parameters. dsGFP was used as RNAi control. The histogram bars represent mean ± SEM of ≥ 3 biological replicates. Bars annotated with the same letter are not significantly different at *p *< 0.05. Panel A: number of eggs laid/BPH; Panel B: Preovipositon Period (days); Panel C: Oviposition period (days); Panel D: Adult female longevity (days).

JGM-treated, dsACC-treated BHP females laid significantly fewer eggs compared to JGM-treated controls (↓77.5%, from 457 to 102.7) and dsGFP treated (↓70.3%, from 345.5 to 102.7) females (Fig. 4A). Similarly, the pre-oviposition period of JGM-treated, dsACC-treated BPH was longer by 3.5 d compared to untreated controls, by 3.8 d in dsGFP-treated controls, and by 4.0 d in JGM-treated controls (Fig. 4B). JGM treatments without dsACC treatment did not influence the pre-oviposition period. The oviposition period of JGM-treated, dsACC-treated BPH was shorter by 7 d compared to JGM-treated BPH and by 9 d to dsGFP-treated BPH (Fig. 4C). dsACC treatment had no influence on adult female longevity (Fig. 4D).

**Figure 5:**
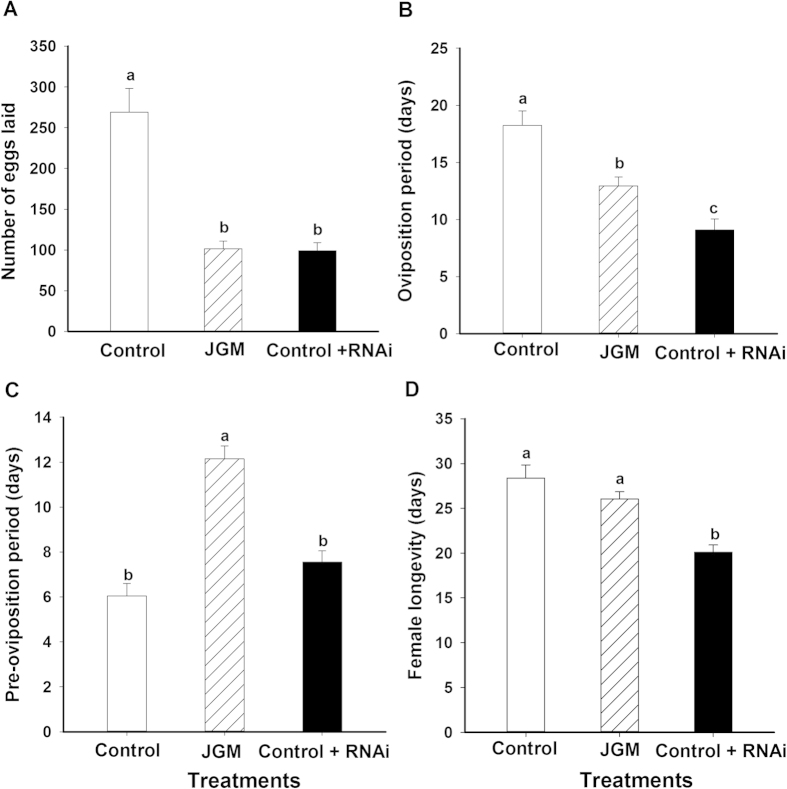
Dietary dsACC influenced WBPH reproduction parameters. dsGFP was used as RNAi control. The histogram bars represent mean ± SEM of  3 biological replicates. Bars annotated with the same letter are not significantly different at *p *< 0.05. Panel (**A**) number of eggs laid/WBPH; Panel (**B**) Oviposition period (days); Panel (**C**) Preovipositon Period (days); Panel (**D**) Adult female longevity (days).

For WBPH, JGM treatment significantly reduced the number of eggs laid by 170 (Fig. 5A) and the oviposition period by 5.1 d (Fig. 5B) compared to controls (without JGM treatment). As expected, dsACC treatment had similar influence on the number of eggs laid and the oviposition period as registered for JGM treatment. JGM treatment delayed the pre-oviposition period by 6.1 d compared to controls; dsACC treatment eliminated the JGM-delayed pre-oviposition period (Fig. 5C). We used control WBPH because the JGM treatments reduced the oviposition period. JGM treatments alone did not influence longevity compared to controls, however, dsACC treatment reduced the longevity (Fig. 5D).

### qPCR analysis of transcription profiles

**Figure 6:**
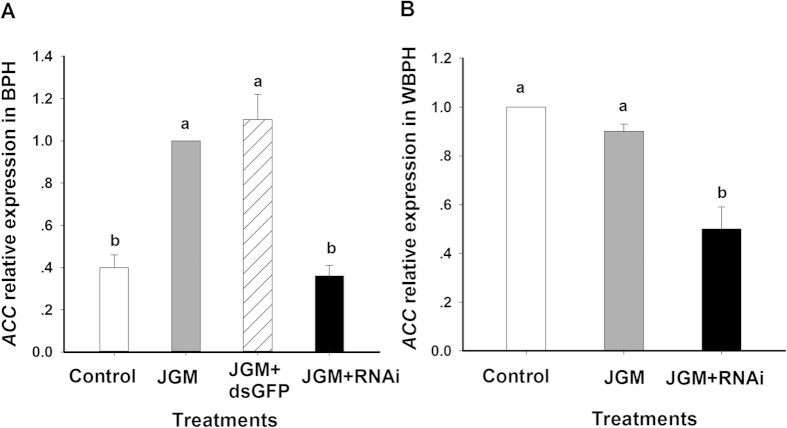
qPCR data for the mRNA expression levels of *ACC* in dsACC-treated BPH and WBPH. dsGFP was used as RNAi control for BPH. The histogram bars represent mean ± SEM of ≽ biological replicates. Bars annotated with the same letter are not significantly at *p *< 0.05. We note WBPH ACC expression was reduced in our transcriptome data (Fig. 3), but not in our qPCR experiment. We ascribed this difference to using different samples and different analytic protocols.

The dietary dsACC treatments led to reduced *ACC* expression in BPH and WBPH. *ACC* expression in control (no JGM, no dsACC) BPH and JGM treated, dsACC*-*treated BPH was compared to JGM treated, dsGFP-treated control groups (*ACC* expression in JGM-treated group = 1.0; Fig. 6A) (Table 2). For WBPH, *ACC* expression in the dsACC*-*treated group was lower compared to the control and JGM-treated group (Fig. 6B).

## Discussion

The data reported in this paper strongly support our hypothesis that long-term exposure to JGM resulted in molecular niche separation (MNS) within the planthopper guild Three key points are germane. First, JGM treatments stimulated BPH reproduction and population growth in field populations, and suppressed the reproduction of WBPH, reducing population growth. Second, transcriptomic analysis identified a panel of differentially expressed genes, of which some were upregulated in JGM-treated BPH, but down-regulated in JGM-treated WBPH, including *ACC*. Third, dsACC treatment eliminated JGM induced fecundity and population growth in BPH. Taken together, these points demonstrate a complete argument for the differential influence of JGM on reproduction of BPH and WBPH and for a likely role of ACC in mediating MNS. To be sure, ACC is not the only protein involved in the JGM effects on planthopper reproduction, however, we are confident ACC is a central mediator because the protein operates at the top of a metabolic pathway^19^. Certainly functional analysis of more genes will be necessary to more fully support our statement.

ACC acts in lipid biosynthesis. It carboxylates acetyl-CoA to generate malonyl-CoA in a biotin-dependent manner, which is then covalently attached to the multifunctional fatty acid synthase (FAS) to synthesize palmitate, a C16 saturated fatty acid^20^. Because ACC acts at the top of a major metabolic pathway, it has potential to influence many aspects of physiology via its influence on fatty acid synthesis. Silencing a key gene in fatty acid synthesis exerts broad and integrated influences on genes in several unrelated pathways. More to the point, fatty acids are among the essential biomolecules for ovarian physiology, including growth, maturation, compact energy storage, and embryonic development. This suggested to us that silencing ACC would influence ovarian development more directly compared to the influence of many other genes acting in reproduction. Palmitate can be converted into a range of additional saturated and mono-unsaturated fatty acids, including palmitoleic acid (16:1), stearic acid (18:0) and oleic acid (18:1). For most, but not all insects, C18 polyunsaturated fatty acids (PUFAs) are essential and are derived from dietary sources. The C18 PUFAs are converted into their C20 counterparts by elongation/desaturation pathways. We demonstrated that JGM-stimulated reproduction in BPH is attended by up-regulation of *ACC*; JGM-suppressed reproduction in WBPH is accompanied by down-regulating *ACC* expression. We infer a mechanistic linkage between reduced *ACC* expression, deficient lipid synthesis and reproduction in planthoppers. For another example of ACC significance, RNAi-medicated *ACC* suppression in mosquitoes, *Aedes aegypti,* led to defective oocytes, which lacked an intact eggshell and gave rise to inviable eggs. ACC suppression leads to reduced lipid biosynthesis and both *ACC-* and *FAS1-*silenced mosquitoes produced fewer eggs than control mosquitoes in the 1^st^ and 2^nd^ gonotrophic cycles^21^.

Generally, responses of intraguild species to environmental stress, such as pesticides, occur in similar trends. For example, the insecticide, triazophos, stimulated reproduction in three members of the planthopper guild, BPH, WBPH and SBPH^22^. Similarly, a sublethal dosage of imidacloprid reduced fecundity of intraguild leafhoppers *Nephotettix virescens* and *N. cincticeps* to one-third and one-half, respectively^23^. Imidacloprid reduced feeding in the aphid species *Myzus persicae* and *Myzus nicotiana*, members of another guild^24^. These similar trends within guilds highlight the differences between BPH and WBPH.

The differential JGM influence on BPH and WBPH has deep significance for rational application of pesticides, a critical need for controlling pests and for mitigating the negative consequences of pesticides in agroecosystems. Populations of BPH, WBPH, and SBPH sequentially peak through the rice growing season, however, during outbreaks of one species, populations of the other planthopper species remain low. If any given pesticides stimulate the reproduction of one member of the planthopper guild, pesticide application may result in environmentally dangerous resurgence of the target or non-target species. It follows that a thorough understanding of pesticide effects on reproduction in all members of the planthopper guild is necessary. We suggest that JGM may be used to control rice sheath blight at the early growth stages of rice (when WBPH, but not BPH, is present). However, the application of JGM should be reduced or delayed when larger BPH populations are present, specifically during the middle and later rice growth stages, to avoid BPH resurgence. Clearly, new knowledge on the influence of other pesticides on the planthopper guild is urgently necessary.

Intraguild species can reduce resource competition through niche partitioning. Here, we introduce MNS, which operates at the level of one or a few genes to reduce niche overlap. In the specific example of the planthopper guild, JGM treatment promoted reproduction of one species and suppressed it in another. Apparently, these two species diverged radically in their adaptions to the presence of JGM in their shared environment. The molecular mechanism of these planthopper adaptations probably involves a single gene, *ACC*. One gene can change in several ways over relatively short time spans, including gene duplication and sequence mutations that influence the functions of the cognate enzymes. The biological significance of MNS may lie in the rapid evolution of a single or a few genes to create a niche separation of larger or smaller dimensions. Although JGM has been present in rice agroecosystems for many planthopper generations, BHP and WBPH may have evolved their differential adaptations to JGM very soon after it was introduced. The continuous use of JGM may have served to maintain the molecular adaptations for decades. We speculate that the suppressive adaptation of WBPH may have allowed the BPH to thrive in the predictable exposure to JGM.

The significance of MNS lies in its explanatory power; it is not limited to planthoppers. MNS may have operated in the adaptive radiation of Darwin’s finches. The group is monophyletic, thought to originate from an ancestral species that arrived in the Galápogos Archipelago from Central or South America. The bird species (they are not actual finches) evolved substantial differences in beak morphology, such as long and pointed beaks in cactus finches and deep, wide beaks in ground finches. These differences reflect differences in feeding niches. In their study of the genes involved in beak development, Abzhanov *et al.* (2006) reported that a single gene encoding calmodulin (a molecule that acts in intracellular calcium signaling) is expressed in higher levels in the developing beaks of cactus finches than in more robust beaks of other species^25^. The rapid radiation of Darwin’s finches, possibly mediated by MNS, allowed separation of feeding niches among the birds.

## Materials and Methods

### Rice variety, insects and pesticide

Seeds of the rice variety Ninjing 1 (japonica rice) were sown in cement tanks (60 × 100 × 100 cm) at the Yangzhou University experimental farm. Thirty-day-old seedlings bearing six leaves were transplanted into pots (35 cm D × 50 cm H) and covered with cages. Four seedlings were planted as a hill in the pots, and each pot contained four hills. Rice plants at the tillering stage were used in the experiments.

BPH were obtained from a stock population maintained at the China National Rice Institute (Hangzhou, China) that was initiated in 2001. WBPH were collected from field populations at the Yangzhou University experimental farm in 2010. They were reared in an insect nursery covered with cages under natural conditions in cement tanks (60 × 60 × 100 cm) from April to October and in a greenhouse in the winter. Prior to the experiments, both colonies were allowed to reproduce for two generations in an insectary (28 ± 2 °C and L16:D8) without insecticide application to ensure sufficient populations for experiments. Technical-grade JGM (C_20_H_35_O_13_N) (61.7%) was supplied by Qianjiang Biochemical Co. Ltd. (Haining, Zhejiang, China).

### JGM influence at the population level

We confirmed the stimulatory and suppressive effects of JGM on planthopper reproduction^22^ in rice field experiments. Next generation nymph numbers were recorded after one generation (about 28–30 days after JGM spray). We conducted JGM foliar sprays with 100 and 200 ppm JGM when 3^rd^–4^th^ instar nymphs reached the peak level in two rice growing regions on 33 × 33 m^2^ plots (Nanning, Guangxi, 22^0^83^′^ N, 108^0^38^′^ E; Changsha, Hunan, 28^0^23^′^ N, 112^0^93^′^ E) in May through August in 2014. Control plants at the same developmental stage as the treated plants were sprayed with tap water and non-active substances (dimethyl sulfoxide and emulsifier) that lacked the effects of insecticides. Each treatment was replicated four times at the Nanning site (with WBPH, no BPH present) and five times at the Changsha site (with BPH, no WBPH). Twenty randomly selected hills were sampled in the center by tapping rice plants at two time points, before foliar spray and at 28–30 days after spray (3rd–4th instar nymphs peak of next generation). The hoppers were transferred into an enamel plate (50 × 50 × 10 cm) and counted. A population growth index (I) was calculated, using: I = Nt + 1/Nt + 0.

### Analysis of JGM-induced gene expression profiles

Since the genome sequence for BPH is available, we analyzed the effect of JGM on differentially expressed genes using digital gene expression profiling (DGE). Because a WBPH genome is not available, we used transcriptome analysis for WBPH. These two approached led to quantitative differences in numbers of differentially expressed genes, seen in Results. One hundred virgin BPH and WBPH females developed from the third instar nymphs treated with JGM and control nymphs (50 adults per group) were placed in a −80 °C freezer for RNA extraction. Our one-sample analysis is a reasonable approach to identifying genes of interest for detailed functional analysis. More to the point, recent reports of sequence studies are based on analysis of one biological sample^26^,^27^. We analyzed gene expression (50 females/group) at 2 days after JGM-treated adult female emergence using DGE. Total RNA was isolated from BPH using the SV Total RNA isolation System (Promega). The samples were treated with DNase, and RNA quantity was evaluated using a spectrophotometer (NanoDrop 2000, Thermo). RNA quality was evaluated using a 2100 Bioanalyzer (Agilent) according to the Illumina instructions.

For BPH, mRNA was isolated form 6 μg of total RNA from each sample using oligo (dT) magnetic beads, and then double-stranded cDNA was synthesized after reverse transcription using oligo (dT) primer bound to magnetic beads. Two restriction enzymes were used to generate sequencing tags. The cDNA was digested with *NlaIII*, then the Illumina adapter 1 was annealed to the 5′ end of the cDNA fragment attached to the magnetic beads. The junction of Illumina adapter I and CATG generates the *MmeI* recognition site. *MmeI* was used to cut the cDNA fragments at 17 bp downstream of the CATG site. After removing the cDNA fraction with magnetic beads, the Illumina adapter 2 was ligated at the 3^′^ end of the tags. After fifteen cycles of linear PCR amplification, 105-bp fragments were purified on 6% TBE PAGE. After denaturation, the single-stranded sequences were fixed onto an Illumina HiSeq TM 2000 chip. Each molecule was grown into a single-molecule cluster sequencing template via *in situ* amplification and four color-coded nucleotides were added to the chip for sequencing by synthesis. Adaptor sequences, low quality tags (tags containing unknown nucleotides N and tags with more than 50% bases of quality value less than 10), empty reads (reads with only 3^′^ adaptor sequences, but no tags), tags that were either too long or too short, and single-copy (probable sequencing error) tags were filtered out to generate clean tags. Sequencing data were evaluated by assessing the distribution of tag expression, the saturation of sequencing data, and experimental reproducibility.

The clean tags were mapped to BPH reference genes at Beijing Genomics Institute (BGI, Shenzhen, China). A preprocessed reference database of all possible CATG + 17 nucleotides tag sequences was created using the reference genes. Subsequently, all clean tags were mapped to the reference database using SOAP2^28^, allowing no more than 1 nucleotide mismatch. Clean tags mapped to multiple genes in the reference sequences were filtered out. The remaining clean tags were unambiguous clean tags. For gene expression analysis, the number of unambiguous clean tags for each gene was calculated, and then normalized to the number of transcripts per million (TPM) clean tags^29^.

We recorded differential gene expression across samples using an algorithm^30^. To compare the differences in gene expression between the samples, the tag frequency in each library was analyzed according to Mortazavi *et al.* (2008)^31^. The false discovery rate (FDR) was used to determine the threshold P value in multiple tests. A FDR ≤ 0.001 and an absolute value of the log2 ratio ≥1 were used as the thresholds to determine significant differences in gene expression. We used the equation


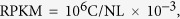


where C = the number of reads that only map to one unigene, N = total number of C, and L = the number of bases in one unigene, to quantify relative gene expression.

*P* value was calculated using GO (http://www.geneontology.org/) with a Bonferoni correction. A corrected *P* value ≤ 0.05 was the threshold for significant enrichment of the gene sets. WEB Gene Ontology Annotation Plot (WEG) software was used for visualizing, comparing and plotting GO annotation results^32^. Pathway enrichment analysis identified significantly enriched metabolic pathways or signal transduction pathways using the KEGG database^33^. Pathways with a *Q* value ≤ 0.05 were taken as significantly enriched pathways.

For WBPH, since there is no genome sequence available, transcriptomic analysis was used to analyze the effect of JGM on differentially expressed genes. Poly(A)^+^ RNA was purified from 10 μg of pooled total RNA using oligo (dT) magnetic beads and fragmented into short sequences in fragmentation buffer. The cleaved poly(A)^+^ RNA was transcribed using random hexamers, and second-strand cDNA was synthesized. After purification using a QIAquick PCR purification Kit, end-repair and ligation of sequencing adaptors, the products were amplified by PCR to create a cDNA library.

Each cDNA library was sequenced by Beijing Genomics Institute (BGI, Shenzhen, China). The raw reads from the images were generated using the Solexa GA pipeline 1.6 and were then cleaned by removal of adaptors sequences, empty reads and low quality reads (including those without adaptors, with an N percentage > 10% or over 50% base quality values Q  10 were filtered). The cleaned reads with an identity of 95% and a coverage length 100 bp were assembled using the SOAP *de novo* software^28^ The resultant contigs were joined into scaffolds using paired-end joining and gap-filling. The scaffolds were clustered to generate unigenes using TGI clustering tools^34^. After clustering, the unigenes were divided into two classes: clusters and singletons.

Unigenes larger than 350 bp were annotated using BLASTx and the GenBank database (http://www.ncbi.nlm.nih.gov), and annotation against protein databases, including non-redundant (nr), SwissPort, COG, KEGG, and GO protein databases, using an E-value cut-off of 10^−5^. For nr annotation, we used Blast2go software (http://www.balst2go.org/) and GO functional classification for all-unigenes using WEGO^30^. After aligning the all-unigenes to the COG database, COG annotations of unigenes were performed using Blastall software against Cluster of Orthologous Groups database. To investigate the metabolic pathway annotation of unigenes, we aligned the all-unigenes to the KEGG database^33^.

### dsRNA synthesis

Based on our reasoning described in Discussion, we targeted *ACC* for RNAi silencing. We analyzed the influence of silencing *ACC* on reproduction in JGM-treated BPH and untreated WBPH (controls).

For dsRNA synthesis, a 479-bp (BPH) or 292-bp (WBPH) fragment with the T7 primer sequence at the 5^′^ ends (Table 1) was amplified. The amplification reactions were programmed for 35 cycles at 95 °C for 1 min, 56 °C or 60 °C for 40 s, 72 °C for 1 min, with a final extension step of 72 °C for 10 min. The identities of PCR products were verified by DNA sequencing. Those sequences in which products from the forward and reverse sequencing aligned well (98–99%) were used for dsRNA synthesis. The green fluorescent protein (GFP) gene (ACY56286) was used as control dsRNA andthe primers GFP-F and GFP-R were used to amplify the GFP fragment (688 bp) (Table 1). dsRNAs were prepared using the T_7_RiboMax Express RNAi System (Promega, Madison, WI, USA). Sense and antisense dsRNAs generated in separate 20 μl total reaction volumes were annealed by mixing both transcription reactions and incubating at 70 °C for 10 min then cooling to room temperature over a 20 min time period. A 2 μl dilution RNase A solution (1:200) and 2 μl RNase-free DNase were added to each mixture solution of both transcription reactions and bathed in 37 °C water for 30 min. The dsRNA was precipitated with 110 μl 95% ethanol and 4.4 μl 3 M sodium acetate (pH5.2), washed with 0.5 ml 70% ethanol and dried at room temperature before dissolving in 50 μL nuclease-free water. The purified dsRNAs were quantified at 260 nm and examined on agarose gels to ensure their integrity.

### *ACC* silencing

Insects were released onto potted rice and treated with JGM as just described. Experimental and control fifth instar nymphs (20/treatment) were collected 3 days after JGM treatments. They were treated with RNAi^33^. dsRNA (0.125 μg/μl; decreased gene expression by 70% in preliminary tests) was used. After four days of feeding on the dsRNA-laced artificial diet, newly emerged females were transferred to culture boxes. A pair from the same replicate were placed in a glass cup containing untreated rice stems for egg-laying and three biological replicates for each treatment were performed. Rice stems were replaced every 2 days. The number of eggs laid on each rice stem was counted under a microscope.

### qPCR analysis

Total RNA from the dsACC-treated and dsGFP-treated control BPHs and WBPHs were extracted and reverse transcribed. Portions (2 μl) of the synthesized first-strand cDNA were amplified by qPCR in 20 μl reaction mixtures using a CFX 96 PCR system (Bio-Rad, California, USA) with this program: 94 °C for 2 min, followed by 40 cycles of 94 °C for 5 s, 49.4 °C (BPH) or 40.3 °C(WBPH) for 30 s, and 72 °C for 30 s. The BPH and WBPH Actin-1 genes were used as reference genes. Primers used for qPCR analysis are listed in Table 1. After amplification, a melting curve analysis was performed in triplicate and the results were averaged. The values were calculated using three independent biological samples and the 2^−△△CT^ method (Livak & Schmittgen 2001)^35^ was used for the analysis of relative *ACC* expression.

### Statistical analysis

We analyzed data on number of eggs laid, pre-oviposition period, oviposition period, adult female longevity for RNAi and population growth after spray in rice field by one-way ANOVA (Table 2). All data are expressed as mean ± SEM, N = at least 3 biological replicates. Multiple comparisons of the means were analyzed using the Fisher-protected least significant difference. All analyses were conducted using the DPS data processing system for practical statistics^36^.

## Additional Information

**How to cite this article**: Zhang, Y.-X. *et al.* RNAi knockdown of acetyl-CoA carboxylase gene eliminates jinggangmycin-enhanced reproduction and population growth in the brown planthopper, *Nilaparvata lugens*. *Sci. Rep.* **5**, 15360; doi: 10.1038/srep15360 (2015).

## Figures and Tables

**Table 1:** PCR primers used in this study.

Primer	Primer sequence
For quantitative real-time PCR	
*QNlACC-F*	5^′^-TTACTGATGGCTTGGCTAC-3^′^
*QNlACC-R*	5^′^-CGACATTACGACCCTGAC-3^′^
*QSfACC-F*	5^′^-TCACCAATCGCCTTACGCT-3^′^
*QSfACC-R*	5^′^-TCGGCTCACAGGGATCAAA-3^′^
For*NlACC* and *SfACC* dsRNA synthesis	
*NlACC-F*	5^′^-TTACTGATGGCTTGGCTAC-3^′^
*NlACC-R*	5^′^-CGACATTACGACCCTGAC-3^′^
*SfACC-F*	5^′^-GAACTGAACGCCTTTGTAGC-3^′^
*SfACC-R*	5^′^-GGGAGGTGGCAGAATGGTA-3^′^
For GFP dsRNA synthesis	
*GFP-F*	5^′^AAGGGCGAGGAGCTGTTCACCG-3^′^
*GFP-R*	5^′^-CAGCAGGACCATGTGATCGCGC-3^′^

**Table 2:** *F*-statistics for all experiments.

Experiment	*F*-Statistic
JGM ↑ BPH populations	*F* = 29.9, df = 2, 12,*P* = 0.0001
JGM ↓ WBPH populations	*F* = 11.8, df = 2, 9,*P* = 0.001
dsACC↓ BHP egg-laying	*F* = 73.4, df = 3, 85,*P* = 0.0001
dsACC↑ BPH pre-oviposition period	*F* = 14.9, df = 3, 89,*P* = 0.0001
dsACC↓ BPH oviposition period	*F* = 9.7, df = 3, 89,*P* = 0.0001
dsACC did not influence BPH longevity	*F* = 1.8, df = 3, 85,*P* = 0.15
dsACC↓ WBPH egg-laying	*F* = 32.2, df = 2, 76,*P* = 0.0001
dsACC↑ WBPH pre-oviposition period	*F* = 26.6, df = 2, 76,*P* = 0.0001
dsACC↓ WBPH oviposition period	*F* = 20.0, df = 2, 76,*P* = 0.0001
dsACC↓ BPH *ACC* expression	*F* = 39.2, df = 3, 8,*P* = 0.0001
dsACC↓ WBPH *ACC* expression	*F* = 31.1, df = 2, 8,*P* = 0.0007
